# Inherited Metabolic Causes of Stroke in Children: Mechanisms, Types, and Management

**DOI:** 10.3389/fneur.2021.633119

**Published:** 2021-03-04

**Authors:** Brahim Tabarki, Wejdan Hakami, Nader Alkhuraish, Kalthoum Graies-Tlili, Marwan Nashabat, Majid Alfadhel

**Affiliations:** ^1^Division of Pediatric Neurology, Department of Pediatrics, Prince Sultan Military Medical City, Riyadh, Saudi Arabia; ^2^Division of Neuroradiology, Department of Radiology, Prince Sultan Military Medical City, Riyadh, Saudi Arabia; ^3^Department of Genetics and Precision Medicine (GPM), King Abdullah Specialized Children's Hospital, King Saud Bin Abdulaziz University for Health Sciences, King Abdulaziz Medical City, Ministry of National Guard Health Affairs, Riyadh, Saudi Arabia; ^4^Medical Genomics Research Department, King Abdullah International Medical Research Center (KAIMRC), King Saud Bin Abdulaziz University for Health Sciences, King Abdulaziz Medical City, Ministry of National Guard Health Affairs, Riyadh, Saudi Arabia

**Keywords:** stroke, stroke-like episodes, mitochondrial diseases, inborn error of metabolism, MRI

## Abstract

A stroke should be considered in cases of neurologic decompensation associated with inherited metabolic disorders. A resultant stroke could be a classical ischemic stroke (vascular stroke) or more commonly a “metabolic stroke.” A metabolic stroke begins with metabolic dysfunctions, usually caused by a stressor, and leads to the rapid onset of prolonged central neurological deficits in the absence of vessel occlusion or rupture. The cardinal features of a metabolic stroke are stroke-like episodes without the confirmation of ischemia in the typical vascular territories, such as that seen in classic thrombotic or embolic strokes. Identifying the underlying cause of a metabolic stroke is essential for prompt and appropriate treatment. This study reviews the major inherited metabolic disorders that predispose patients to pediatric stroke, with an emphasis on the underlying mechanisms, types, and management.

## Introduction

Pediatric stroke is surprisingly common, affecting 25 in 100,000 newborns and 12 in 100,000 children (1). Stroke is classically divided in two types—ischemic and hemorrhagic—and characterized as a neurological deficit attributable to an acute focal injury of the central nervous system due to a vascular cause. The most relevant risk factors and causes of a pediatric stroke are vasculopathies, infections, cardiac anomalies, or coagulopathies (1). Although strokes are rarely caused by metabolic disorders, they are increasingly recognized as inherited causes of pediatric strokes (2–7). Metabolic disorders may cause classical ischemic strokes, such as those occurring in patients with cystathionine β-synthase deficiency, or more commonly “metabolic strokes,” such as those occurring in patients with mitochondrial disorders (3–5). Metabolic strokes begin with metabolic dysfunctions that lead to the rapid onset of central neurological deficits in the absence of vessel occlusion or rupture (3–5, 7). Early recognition of these metabolic disorders and prompt initiation of appropriate treatment may improve the prognosis. This review provides an overview of various metabolic strokes occurring in children, with an emphasis on their underlying mechanisms, type, and management.

## Methods

The online database MEDLINE was used to search for literature published between January 1985 and December 2019, without any date or language restrictions. We used a combination of the following search terms for literature review: “children AND stroke-like episode (SLE)” (430 papers), “children AND stroke-like lesions” (82 papers), “children AND stroke AND inborn error of metabolism” (609 papers), and “children AND stroke AND mitochondrial” (545 papers). We independently reviewed the articles to identify inherited metabolic and mitochondrial causes of stroke in pediatric patients and systematically screened titles, abstracts, and full texts of the retrieved publications. Reviews and editorials were excluded (220 papers).

The common, pediatric onset, inherited metabolic causes of stroke are outlined in Tables 1–3.

**Table 1:** Common mitochondrial disorders associated with stroke.

**Condition**	**Gene**	**Treatment**
MELAS (8)	*MTTL1 (>80%)* *Other mtDNA*	L-arginine 500 mg/kg (IV), 150 to 300 mg/kg/day oral L-carnitine: 100 mg/kg/d L-citrulline: 500 mg/kg/d
Leigh syndrome (9)	Up to 75 monogenic genes (mtDNA or nDNA)	Supportive care^*^
MERRF (10)	*MT-TK*	Supportive care
Chronic progressive external ophthalmoplegia (2)	C10orf2	Supportive care
Leber's hereditary optic neuropathy (2)	ND6	Idebenone 900 mg/day
POLG1-related (11)	POLG1	Supportive care
Triple-H syndrome (12)	SLC25A15	Low protein diet Supplementation: arginine, citrulline, ornithine Ammonia scavengers
CoQ-deficiency (13)	9 genes encoding proteins directly involved in the synthesis of coenzyme Q_10_	CoQ_10_ supplementation:5–50 mg/kg/day, orally
Mitochondrial multiorgan disorder syndrome (2)	*Twinkle*	Supportive care
OPA1-related (14)	*OPA1*	Supportive care
3-ketothiolase deficiency (4, 5)	*ACAT1*	Mildly restricted protein intake Avoidance of fat-rich diet L-carnintin
FBXL4-related (2)	*Fbxl4*	Supportive care
Complex III deficiency (2)	*TTC19*	Supportive care

* *Supportive care include fluid support, metabolic support, and treatment of the underlying triggers*.

**Table 2:** Common inherited metabolic disorders associated with increased risk of stroke.

**Disease**	**Gene**	**Treatment**
**Metabolic stroke**		
Propionic academia (15)	*PCCA/PCCB*	Dialysis for metabolite toxicity (acute), Protein restriction diet (chronic)
Glutaric aciduria type 1 (16)	*GCDH*	Lysine/tryptophan-restricted diet L-carnitine supplementation Riboflavin supplementation
Glutaric aciduria type 2 (5)	*ETF-A, ETF-B, ETFDH*	High-carbohydrate diet L-carnitine supplementation
Isovaleric aciduria (17)	*IVD*	Moderate restriction of proteins Oral administration of glycine and L-carnitine
Methylmalonic aciduria (18)	>60% *MMUT*	Low-protein diet Hydroxocobalamin injections for cobalamin-responsive patients L-carnitine supplementation High energy diet
Carbamoyl phosphate synthetase I deficiency (19)	*CPS1*	Dialysis as acute treatment Low protein diet Ammonia scavenger therapy
Ornithine transcarbamylase deficiency (20)	*OTC*	Dialysis as acute treatment Low protein diet Ammonia scavenger therapy
Citrullinemia (21)	*ASS1, SLC25A13*	Dialysis as acute treatment Low protein diet Ammonia scavenger therapy
Congenital disorder of glycosylation (1a) (22)	*PMM2*	Supportive care Acetazolamide
Canavan disease (4, 5)	*ASPA*	Supportive care
**Ischemic stroke**		
Cerebrotendinous xanthomatosis (23)	*CYP27A1*	Chenodesoxycholic acid 750 mg/day (oral)
Homocysteinuria (24)	*CBS*	Restrict methionine, supplement with folate, cobalamin, betaine, and pyridoxine
Fabry disease (25)	*GLA*	Enzyme replacement therapy
Cystinosis (26)	*CTNS*	Cystine depleting agent (cysteamine) Renal replacement therapy Hormonal therapy
Pompe disease (27)	*GAA*	Enzyme replacement therapy

**Table 3:** Risk factors for inherited metabolic stroke classified according to the age group.

	**Newborns**	**Toddlers**	**Teenagers**
**Ischemic stroke**			
MTHFR	++	++	++
Fabry disease			+++
Pompe disease			++
Cerebrotendinous xanthomatosis			+++
Homocysteinuria	++	++	++
Cystinosis			++
**Metabolic stroke**			
Propionic academia		+++	++
Glutaric aciduria		+++	
Isovaleric aciduria		++	
Methylmalonic aciduria	++	++	++
Carbamoyl phosphate synthetase I deficiency		++	+
Ornithine transcarbamylase deficiency	++	++	++
Citrullinemia	+	++	
Congenital disorder of glycosylation (1a)		+++	+
Canavan disease		++	
MELAS		++	+++
Leigh syndrome			
MERRF		++	++
Chronic progressive external ophthalmoplegia		+	++
Leber's hereditary optic neuropathy			++
POLG1-related		+++	+
Triple-H syndrome		++	
CoQ-deficiency		++	+
Mitochondrial multiorgan disorder syndrome		+	++
OPA1-related			++
3-ketothiolase deficiency		+++	
FBXL4-related		++	
Complex III deficiency		++	+

*MTHFR, Methylene-tetrahydrofolate reductase*.

*+ + +, common; + +, occasional; +, rare*.

## Metabolic Stroke

### Mitochondrial Disorders and Stroke

Mitochondrial disorders usually have a multisystem presentation and are among the most common forms of inherited neurological disorders (6). Patients with mitochondrial disorders are at risk of developing metabolic stroke (6, 7). SLEs are the hallmark of mitochondrial encephalopathy with lactic acidosis and stroke-like episode (MELAS) syndrome, and they appear less frequently in other specific or non-specific mitochondrial disorders (2–14, 28–35). Although the pathophysiology of SLEs is not entirely clear, it may involve mitochondrial dysfunction in the neurons and capillary endothelial cells (2, 7). The vascular theory emphasizes the potential role of mitochondrial proliferation in the smooth muscle layer of the small arteries and arterioles, which impairs autoregulation, and results in ischemia and the development of stroke-like lesions. In addition, several studies have reported a link between nitric oxide deficiency, low plasma levels of arginine and citrulline, and the pathogenesis of SLEs (2, 7). A more recently published study reported that severe mitochondrial complex I defects and preferential loss of inhibitory inter-neurons could potentially lead to neuronal hyper-excitability (28). Table 1 lists the common mitochondrial disorders that may predispose patients to metabolic stroke.

#### Disorders of Mitochondrial DNA

**Figure 1:**
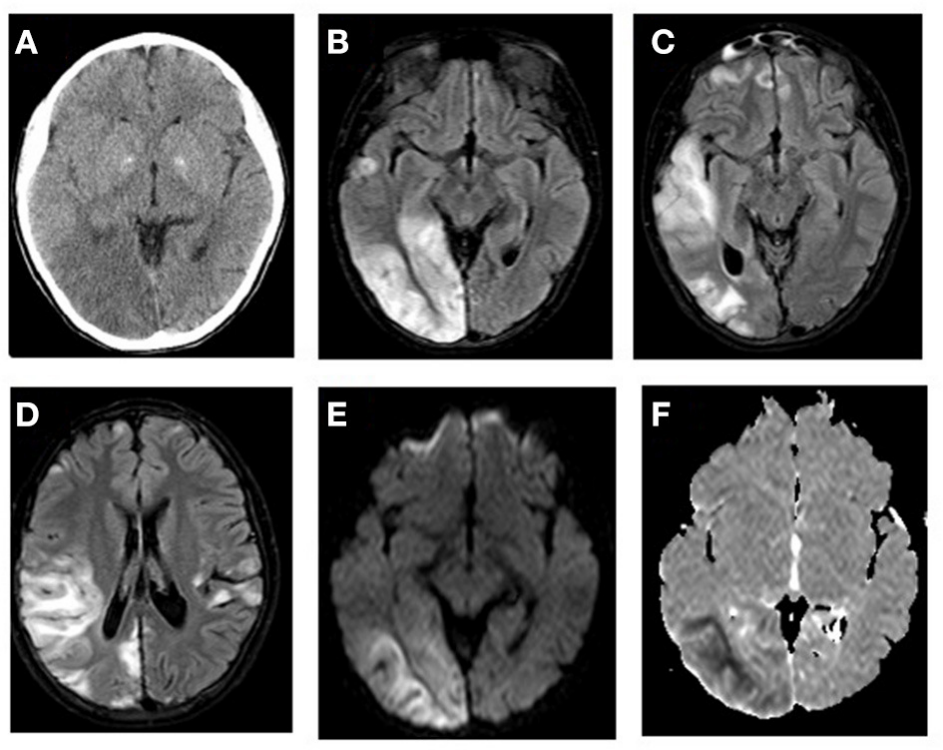
*MTTL1*-related MELAS with stroke-like episodes. **(A)** CT scan shows hypodense areas in the right posterior temporal and occipital lobes with mass effect and effacement of the sulci on the surface of right cerebral hemisphere. FLAIR MR images **(B–D)** demonstrates bilateral multifocal cortical and subcortical white matter predominantly in the right temporo-occipital areas with swollen gyri. The lesions showed increased signal on diffusion weighted image **(E)** and low signal on ADC map **(F)** of the subcortical white matter related to the acute ischemic insult.

**Figure 2:**
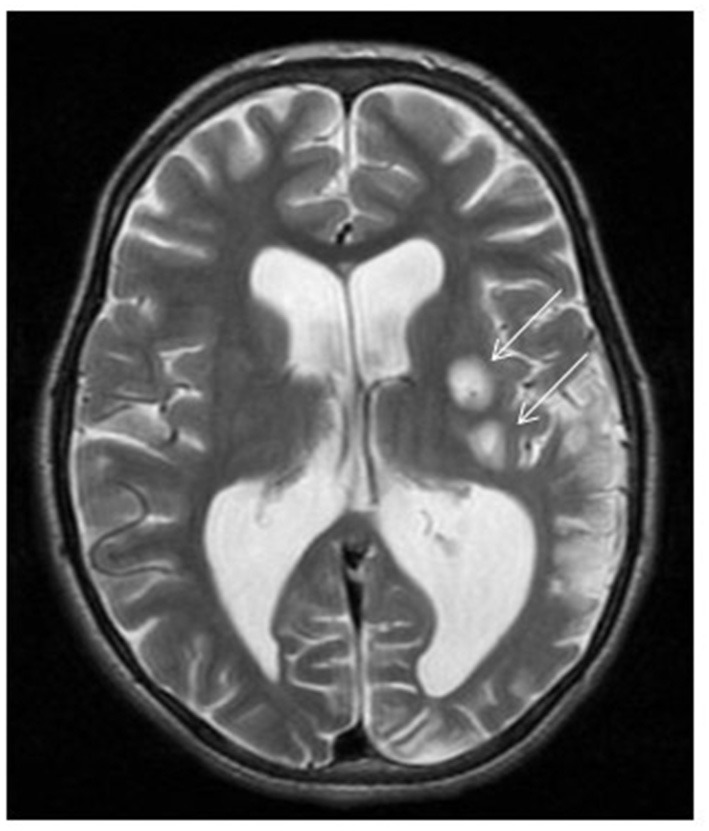
*ND5*-related MELAS with stroke-like episodes. Brain MRI demonstrates two foci of abnormal high T2 signal intensity involving the left putamina (arrows) with restricted diffusion (not showed) representing acute insults. Left parieto-occipital area of encephalomalacia with paucity of the white matter. Mild ventriculomegaly more pronounced at left trigone due to ex vacuo dilatation.

MELAS is a prototypical mitochondrial disorder that presents with acute metabolic stroke. In ~80% of cases, MELAS is associated with a point mutation in the *MTTL1* gene encoding tRNA-Leu (m.3243A>G). In the remaining cases, MELAS is caused by other variants of *MTTL1* or other mitochondrial gene mutations (8). Accumulating evidence suggests that SLEs occur in MELAS partly due to impaired vasodilation secondary to aberrant nitric oxide metabolism. Endothelium-dependent vascular relaxation is mediated by nitric oxide metabolism, a process in which arginine plays a crucial role. Patients with MELAS have low levels of arginine during the acute phase of their SLEs. The lack of arginine is theorized to prevent nitric oxide production, leading to vasoconstriction, hypoxemia, and eventually resulting in visible SLEs on imaging. SLE lesions seen in patients with MELAS are typically not restricted to a vascular territory (2, 7). Clinically, the presentation of SLEs includes migraine or migraine-like headaches, encephalopathy, visual field defects, seizures, or vomiting. Seizures, which are usually difficult to control in MELAS, increase metabolic demand, aggravate stress, and extend the injured area. Neuroimaging assessments have shown that brain parenchymal changes in stroke-like lesions are typically localized in the occipital, parietal, or temporal lobes, and occur in ~90% of patients with MELAS (Figures 1, 2); however, such changes can also occur in any other brain regions (8, 29, 30). Interestingly, SLE is a poor prognosticator in patients with MELAS. Zhang et al. recently reported that in 138 patients with MELAS, SLE was an independent risk factor for death (31).

Acute treatment of SLEs in MELAS is initially based on aggressive management of stressors, such as infections or seizures. The patients should also be administered with intravenous arginine and normal saline boluses to maintain cerebral perfusion, in addition to dextrose-containing fluids to reverse the ongoing or impending catabolism (29). Chronic management of MELAS with appropriate antiseizure medications and infection prophylaxis remains the best approach to prevent SLEs. Recent reports have highlighted the role of taurine supplementation in preventing metabolic decompensation (30).

#### Disorders of Nuclear DNA

Patients with almost any form of mitochondrial disease, including those with a nuclear genetic etiology, may develop SLEs (2). The most common SLEs, but rare disease forms, are SLEs associated with variants of DNA polymerase γ (*POLG1*), a gene that is classically linked to intractable epilepsy in Alpers syndrome (11). The fact that SLEs universally occur during metabolic decompensation underscores the importance of aggressive fluid and glucose management and the avoidance of any drugs that are toxic to the mitochondria and may precipitate iatrogenic SLEs.

#### Leigh Syndrome

**Figure 3:**
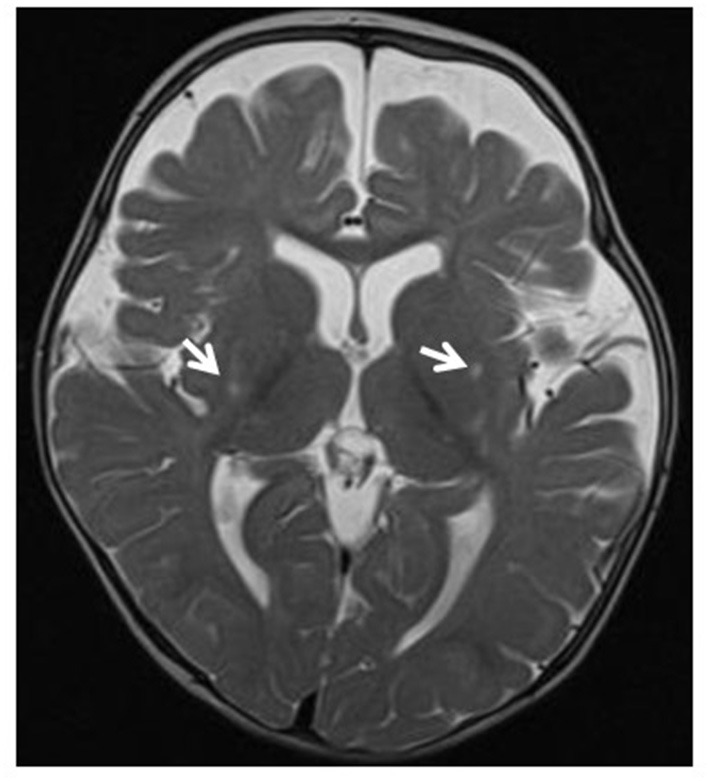
Leigh syndrome. Tiny foci of abnormal T2 signal intensity seen involving both putamina (arrows). Enlarged anterior extra-axial CSF spaces with paucity of the deep frontal white matter indicating moderate atrophic changes.

**Figure 4:**
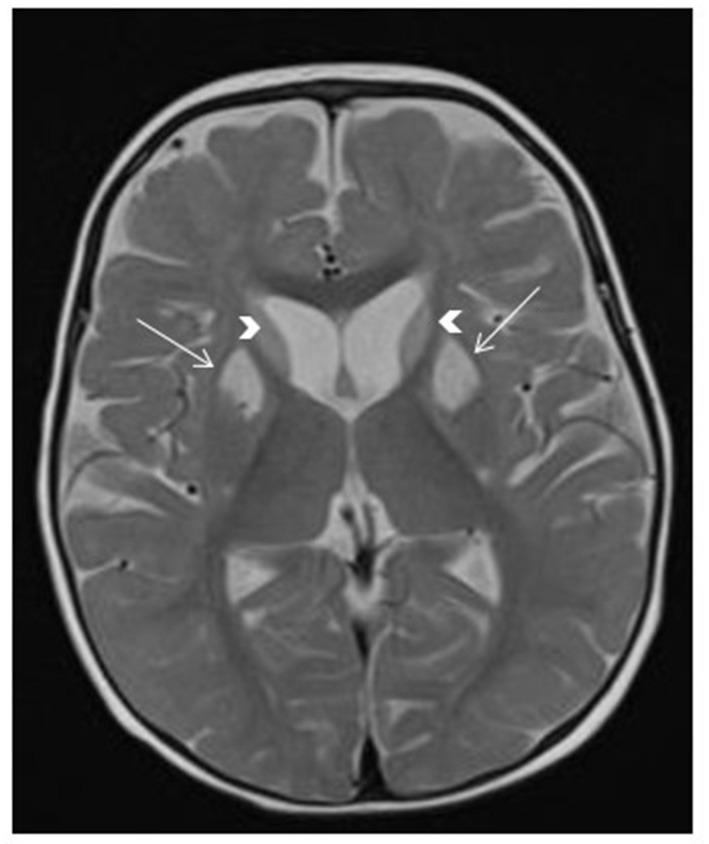
*SERAC1*-related 3-methylglutaconic aciduria with deafness, encephalopathy and Leigh-like syndrome (MEGDEL). Brain MRI shows T2 hyperintensity in the bilateral caudate nucleus (arrowheads) and bilateral anterior putamen (arrows), associated with mildly enlarged anterior subarachnoid spaces.

Leigh syndrome, the most common form of pediatric mitochondrial disease, is characterized by psychomotor regression, seizures, respiratory failure, and lactic acidosis (6). Neuroimaging findings of patients with this disease include bilateral symmetrical lesions in the basal ganglia, diencephalon, brainstem nuclei, cerebellum, and spinal cord (Figures 3, 4). Leigh syndrome, although genetically heterogenous, is most often caused by pathogenic variants of the nuclear genes inherited in an autosomal recessive pattern. More than 75 disease-associated mitochondrial DNA (mtDNA) and nuclear DNA mutations have been identified in patients with Leigh syndrome (9, 10, 32). In a multicenter study of 130 patients with Leigh syndrome, 4% of the patients were found to have developed SLEs (32). Treatment of Leigh syndrome is mainly supportive. Fluid and metabolic support as well as treatment of underlying triggers are crucial during acute neurologic injury.

### Other Inherited Metabolic Diseases and Stroke

The majority of inborn errors of metabolism cause brain injury. Metabolic stroke and SLEs are manifestations of these injuries (15–27, 36–42). The most common inborn errors of metabolism that potentially predispose patients to metabolic stroke are outlined in Table 2.

#### Propionic Acidemia

**Figure 5:**
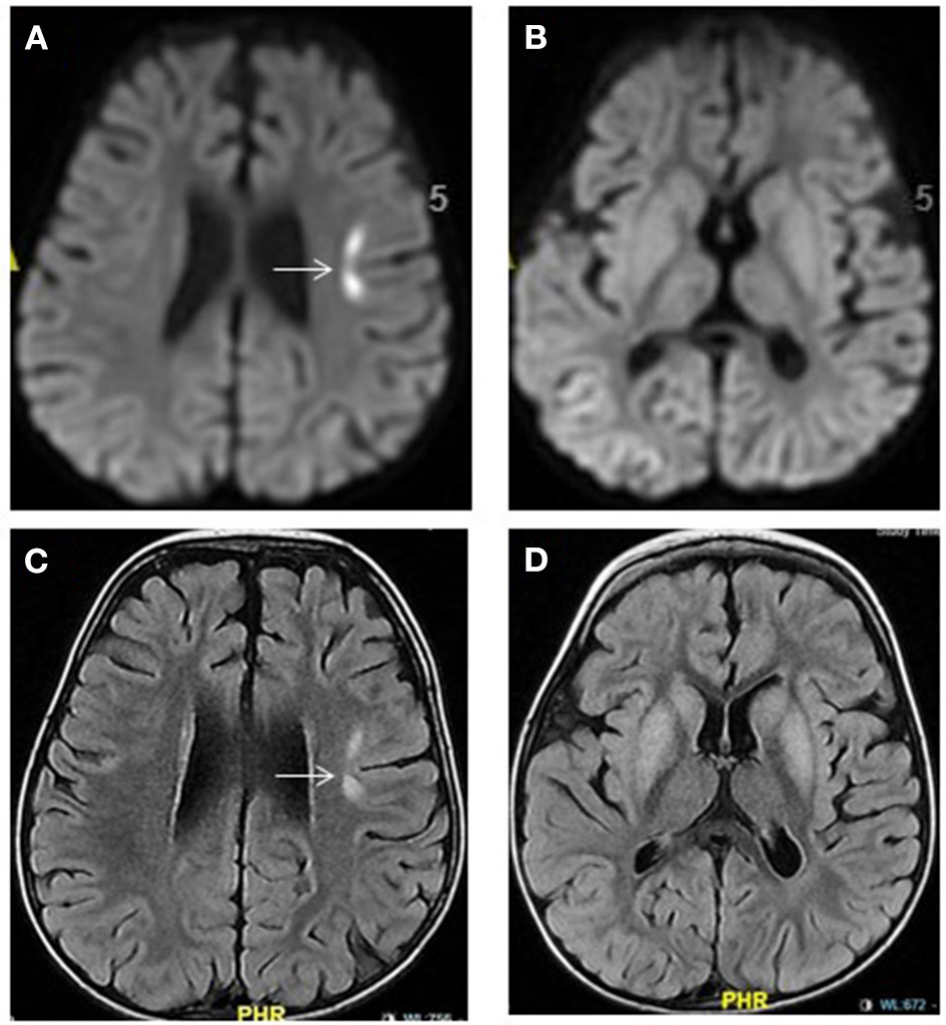
Propionic acidemia. Brain MRI: Axial diffusion-weighted imaging **(A,B)** and FLAIR images **(C,D)** show hyperintensities in the bilateral basal ganglia and in the left frontal cortical-subcortical focal area (arrow). The latter showing diffusion restriction **(A)** representing acute injury. No diffusion restriction within the basal ganglia seen **(B)**.

Propionic acidemia, an autosomal recessive disorder, is caused by defects in propionyl CoA carboxylase, a key enzyme responsible for amino acid catabolism. The clinical features of this condition include lethargy, vomiting, poor feeding, and hypotonia. Metabolic decompensation may present as a focal neurologic deficit or as a change in mental status suggestive of a stroke (15, 36, 37). The most typical imaging findings in propionic academia are bilateral basal ganglia lesions, and these lesions may also involve the cortex or subcortical white matter (Figure 5). SLEs are increasingly reported in patients with propionic academia that can manifest with altered mental status, neurological deficits, and movement disorders (15, 36, 37). Like other metabolic conditions, the deep gray structures, particularly the basal ganglia, appear uniquely susceptible and vulnerable to the effects of propionic acidemia, both during and in between the periods of metabolic compromise. The primary treatment for propionic acidemia includes fluid therapy, electrolyte balance, and if needed, dialysis.

#### PMM2-CDG (Congenital Disorder of Glycosylation Type 1a)

PMM2-CDG is the most common congenital glycosylation disorder. SLEs are one of the acute neurological complications that may present with PMM2-CDG and are observed in ~15–55% of patients with this disease (22). SLEs are typically triggered by stressors, such as infections or head trauma. In patients with PMM2-CDG, SLEs are characterized by a loss of consciousness, neurological deficits, and, occasionally, seizures. Interestingly, acute brain injury is not detectable on magnetic resonance imaging (MRI) in the majority of patients with PMM2-CDG (22, 38, 39). Multiple underlying mechanisms have been suggested to explain the pathophysiology of SLEs in PMM2-CDG, including hypoperfusion, ischemia, and abnormal CaV2.1 function due to aberrant N-glycosylation (38). Although only supportive treatment is currently provided to patients with PMM2-CDG, acetazolamide has been shown to be well-tolerated and effective for cerebellar motor syndrome caused by PMM2-CDG, and its ability to prevent SLEs is currently being studied (39).

#### Glutaric Aciduria

**Figure 6:**
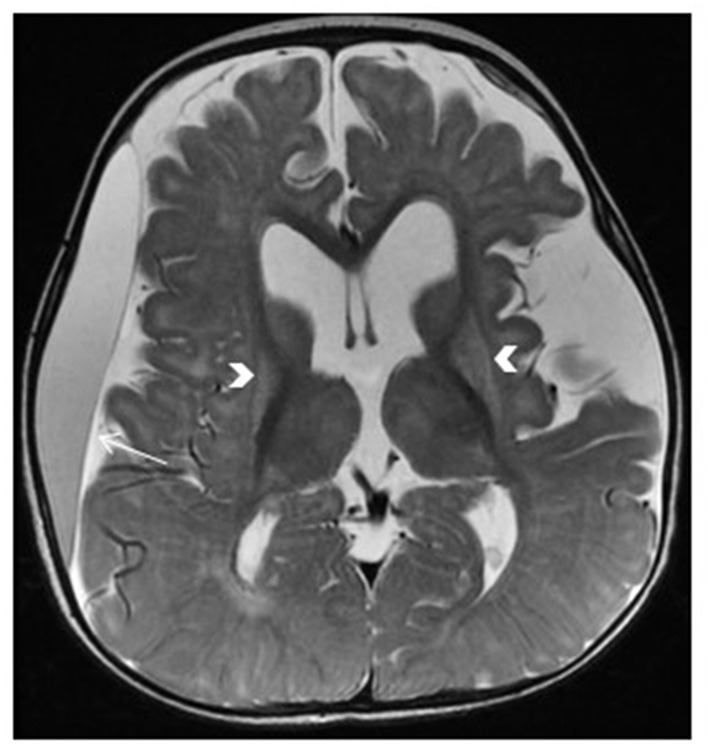
Glutaric aciduria type 1. Brain MRI: axial T2-Weighted image demonstrates bilateral abnormal hyperintense signal and atrophic changes of the putamina (arrows), enlarged subarachnoid spaces, dilated lateral ventricles, and subdural effusion predominantly on the right side (arrow).

Glutaric aciduria type 1, an autosomal recessive disorder, is caused by mutations in the GCDH gene and characterized by macrocephaly with dystonia, which may present with intermittent periods of severe metabolic decompensation resulting in encephalopathy, and seizures (5). The striatum is particularly at risk in patients with glutaric aciduria type 1 and undergoes necrotic changes that are notable on MRI (Figure 6). Retrospective studies have suggested that the selective vulnerability of the striatum may be evident on imaging even before the onset of clinical symptoms (42). The pathophysiology of a metabolic stroke in glutaric aciduria is multifactorial and includes neuronal vacuolization and swelling, secondary ischemia associated with mitochondrial failure, and impingement of brain capillaries. Dietary management is crucial, including avoidance of lysine- and tryptophan-containing foods and supplementation of L-carnitine and riboflavin.

Glutaric aciduria type 2 is an autosomal recessive disorder affecting fatty acid β-oxidation and amino acid metabolism; it is caused by impaired assembly of the electron transfer flavoprotein. It is significantly rarer than the type 1 disease, although strokes have also been reported in children with this form of glutaric aciduria. The treatment of glutaric aciduria type 2 is empirical, consisting of a high-carbohydrate diet and L-carnitine supplementation (5, 6).

### Ischemic Stroke

#### Homocystinuria

**Figure 7:**
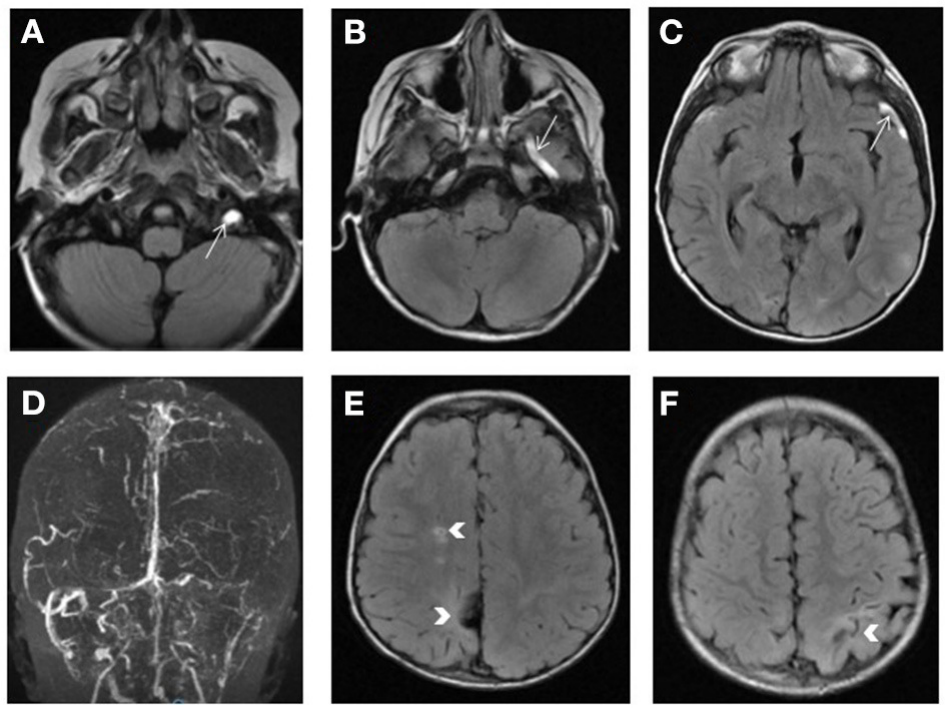
Homocystinuria. Bright signal of the left transverse and sigmoid sinuses as well as the left jugular vein, superficial middle cerebral vein (arrow) and vein of Labbe on FLAIR weighted images **(A–C)** consistent with venous thrombosis and confirmed by the loss of the normal flow signal on 2D TOF venogram **(D)**. Bilateral areas of encephalomalacia involving the parietal lobes with lacunar infarct in the right centrum semiovale (arrows in **E,F**).

In homocystinuria, elevation of plasma homocysteine and methionine levels results in a predisposition to thromboembolic events. The mechanisms underlying vascular disease and thrombosis are multifactorial (24, 40). Elevated plasma homocysteine concentrations have been associated with increased blood coagulation, increased cholesterol synthesis, increased oxidative stress, reduced apolipoprotein A1 synthesis leading to reduced concentrations of high-density lipoprotein, endothelial cell damage, and smooth muscle cell proliferation (24, 40). Homocystinuria, a rare inherited disorder affecting the methionine catabolism pathway, is primarily caused by cystathionine beta-synthase deficiency, which impairs cystathionine synthesis and leads to the accumulation of homocysteine. The clinical presentation of the disease includes abnormalities of the eye, skeletal, nervous, and vascular system. Up to 32% of untreated individuals with cystathionine beta-synthase deficiency will experience thromboembolic episodes, involving both the large and small vessels of the brain (Figure 7), before 20 years of age. In contrast to most of the inherited metabolic and mitochondrial diseases causing SLEs, homocystinuria is a cause of a true ischemic stroke (24, 40). Patients with cystathionine beta-synthase deficiency generally require treatment with a low-methionine diet and/or betaine, folate, cobalamin, and pyridoxine.

Another inherited cause of hyperhomocysteinemia is methylenetetrahydrofolate reductase deficiency, a known risk factor for vascular stroke.

#### Fabry Disease

Fabry disease, an X-linked lysosomal disease, is characterized by the accumulation of globotriaosylceramide and its deacylated form globotriaosylsphingosine (Lyso-Gb3) in the cells of various organ systems (4). The multisystemic effects of this disease include renal failure, cardiac complications, and cerebrovascular disease. Stroke, caused by cerebral vasculopathy, is the most frequent and severe clinical event in patients with Fabry disease (4). A study that analyzed 2,446 patients in the Fabry Registry reported that stroke occurred in 6.9% of men and 4.3% of women (25). MRI findings of patients with Fabry disease usually demonstrate extensive white matter lesions at baseline, and individuals with a new-onset, acute neurologic deficit may only demonstrate the progression of white matter lesions. Although subtle, new imaging findings may appear in some cases, large new asymmetric lesions are rare (4). Patients with Fabry disease should receive prompt enzyme replacement therapy before they incur irreversible damage caused by heart failure, renal fibrosis, or stroke. Acute therapy should focus on stabilizing metabolism, along with fluid and electrolyte management.

## Discussion

Inherited metabolic and genetic defects increase the risk of a stroke (3–5). The physiopathology of a metabolic stroke in patients with inherited metabolic diseases are overlapping (2). Metabolic disorders may cause thromboembolic events in people without other typical ischemic stroke-associated risk factors (4, 5). However, in certain metabolic conditions, cellular energy failure, with consequent cell death, can occur without the cessation of blood flow. The pattern of cell death typically does not follow the classical vascular distribution because metabolic stoke is not a disorder caused by impaired blood flow; however, it may be more diffuse or random (2, 7, 8). However, similar to that in thromboembolic stroke, these metabolic crises triggering cellular failure also require reversals to prevent increased or permanent cell death. Furthermore, reduction of aggressive stressors, and in selected cases, specific therapies (i.e., arginine supplementation for MELAS) should have the same urgency as alteplase administration for the treatment of a classical stroke (2, 8). The recognition of childhood stroke cases with an inherited metabolic basis is an important step in understanding relevant disease mechanisms as well as identifying appropriate management and prevention procedures. The following elements in a patient's medical history and neuroimaging findings provide important indications that can alert physicians of possible underlying metabolic disorders presenting as a stroke: no prior history of risk factors leading to a stroke, such as cardiac anomalies, hematological disorders, coagulation disorders, infections, or trauma; a positive family history of stroke; recurrent stroke; young age; unusual systemic manifestations, such as vomiting, failure to thrive; abnormal neurological findings, such as migraine-like headache, weakness, depressed mental status, visual deficits, or seizures; involvement of other systems (eye, skin, and kidneys); metabolic acidosis or high lactic acid levels; and/or neuroimaging findings, such as stroke-like lesions or lesions characterized by a particular metabolic disorder (1–4, 7). SLEs are better visualized by multimodal MRI; although they present as vasogenic edema, SLEs may also include cytotoxic components. Perfusion studies may show hyperperfusion in the acute stage of stroke-like lesions (2).

Conversely, in patients known to have a condition that may predispose them to strokes (e.g., mitochondrial disorders or other inborn errors of metabolism), close monitoring is needed during acute decompensation triggered by stressful events. Metabolic stroke should be included in the differential diagnosis of any acute neurological deficits or presentation of altered consciousness levels.

## Conclusion

Metabolic stroke is a high-morbidity complication of many inherited metabolic disorders. Physicians should maintain a high index of suspicion for SLEs in patients with metabolic disorders presenting a decreased level of consciousness, acute neurologic deficits, or seizures, especially in the presence of fever or head trauma. Ischemic strokes are also complications of inherited metabolic disorders and should be included in the relevant differential diagnoses. Early recognition, prompt and appropriate metabolic support, and supportive care are necessary to minimize long-term sequelae of metabolic stroke. Clinicians' familiarity with the differences between stroke subtypes associated with specific diseases may facilitate rapid diagnosis and treatment. Long-term prevention is directly related to disease-specific treatments.

## Data Availability Statement

The original contributions presented in the study are included in the article/Supplementary Material, further inquiries can be directed to the corresponding author/s.

## Author Contributions

All authors participated in gathering the data, designing the article, and discussing and editing the manuscript.

## Conflict of Interest

The authors declare that the research was conducted in the absence of any commercial or financial relationships that could be construed as a potential conflict of interest.
